# Superior vena cava isolation using a pentaspline pulsed-field ablation catheter: feasibility and safety in patients undergoing atrial fibrillation catheter ablation

**DOI:** 10.1093/europace/euae160

**Published:** 2024-06-14

**Authors:** Pierre Ollitrault, Corentin Chaumont, Jonaz Font, Martin Manninger, Sergio Conti, Paweł T Matusik, Bart A Mulder, Virginie Ferchaud, Arnaud Pellissier, Mayane Al Khoury, Paul Milliez, Laure Champ-Rigot, Frédéric Anselme

**Affiliations:** Electrophysiology Unit, Department of Cardiology, Regional University Hospital, Avenue de la Côte de Nacre, 14000 Caen, France; Department of Cardiology, Rouen University Medical Center, Rue de Germont, 76031 Rouen, France; Electrophysiology Unit, Department of Cardiology, Regional University Hospital, Avenue de la Côte de Nacre, 14000 Caen, France; Department of Cardiology, Pôle de Formation et de Recherche en Santé, Rue des Rochambelles, 14000 Caen, France; Division of Cardiology, Department of Internal Medical, Graz University Medical Center, Graz, Austria; Department of Cardiology, ARNAS Civico Hospital, Palermo, Italy; Department of Cardiology, St. John Paul II Hospital, Prądnicka 80, 31-202 Kraków, Poland; Institute of Cardiology, Faculty of Medicine, Jagiellonian University Medical College, Prądnicka 80, 31-202 Kraków, Poland; Department of Cardiology, Groningen University Medical Center, Groningen, The Netherlands; Electrophysiology Unit, Department of Cardiology, Regional University Hospital, Avenue de la Côte de Nacre, 14000 Caen, France; Electrophysiology Unit, Department of Cardiology, Regional University Hospital, Avenue de la Côte de Nacre, 14000 Caen, France; Electrophysiology Unit, Department of Cardiology, Regional University Hospital, Avenue de la Côte de Nacre, 14000 Caen, France; Electrophysiology Unit, Department of Cardiology, Regional University Hospital, Avenue de la Côte de Nacre, 14000 Caen, France; Department of Cardiology, Pôle de Formation et de Recherche en Santé, Rue des Rochambelles, 14000 Caen, France; Electrophysiology Unit, Department of Cardiology, Regional University Hospital, Avenue de la Côte de Nacre, 14000 Caen, France; Department of Cardiology, Rouen University Medical Center, Rue de Germont, 76031 Rouen, France

**Keywords:** Pulsed-field ablation, Catheter ablation, Atrial fibrillation, Superior vena cava, Feasibility, Outcome

## Abstract

**Aims:**

Superior vena cava (SVC) isolation during atrial fibrillation catheter ablation is limited by the risk of collateral damage to the sinus node and/or the phrenic nerve. Due to its tissue-specificity, we hypothesized the feasibility and safety of pulsed-field ablation (PFA)–based SVC isolation.

**Methods and results:**

One hundred and five consecutive patients undergoing PFA-based AF catheter ablation were prospectively included. After pulmonary vein isolation (±posterior wall isolation and electrical cardioversion), SVC isolation was performed using a standardized workflow. Acute SVC isolation was achieved in 105/105 (100%) patients after 6 ± 1 applications. Transient phrenic nerve stunning occurred in 67/105 (64%) patients but without phrenic nerve palsy at the end of the procedure and at hospital discharge. Transient high-degree sinus node dysfunction occurred in 5/105 (4.7%) patients, with no recurrence at the end of the procedure and until discharge. At the 3-month follow-up visit, no complication occurred.

**Conclusion:**

SVC isolation using a pentaspline PFA catheter is feasible and safe.

## Graphical Abstract

**Graphical Abstract:**
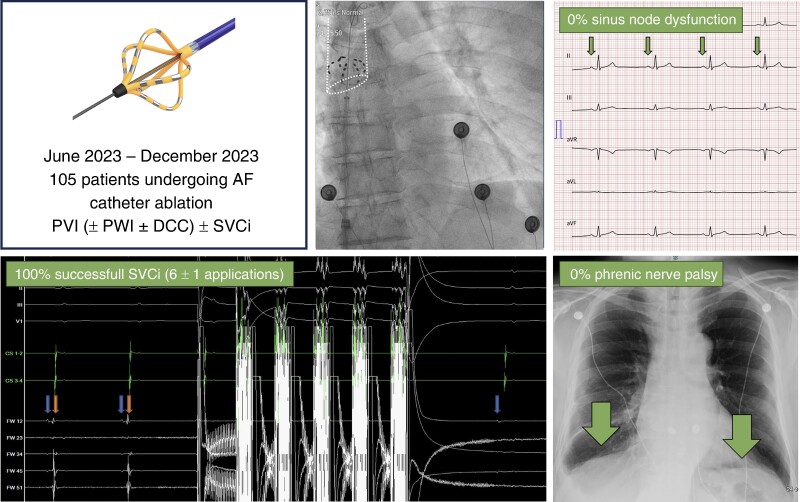

**What’s new?**

- Superior vena cava (SVC) isolation during atrial fibrillation (AF) catheter ablation might be limited by the risk of collateral damage to adjacent structures (phrenic nerve and sinus node). Pulsed-field ablation (PFA) might overcome those limitations.
- SVC isolation using a pentaspline PFA catheter is feasible, with 100% SVC isolation after first PFA application.
- Even though transient phrenic nerve stunning occurred in 64% of patients, no complication regarding phrenic nerve (or sinus node function) occurred at discharge and during follow-up.
- The impact of PFA-based SVC isolation on AF catheter ablation efficacy needs to be evaluated by a dedicated randomized controlled trial.

## Introduction

Pulsed-field ablation (PFA) is a novel, non-thermal, energy allowing fast, efficient, and safe pulmonary vein isolation (PVI) in patients undergoing atrial fibrillation (AF) catheter ablation.^1,2^ Some authors suggested that PFA could also be used to perform additional lesion sets, such as posterior wall isolation (PWI) or mitral isthmus line,^3,4^ but evidence is still limited.

Superior vena cava (SVC) is known to be a location of non–pulmonary vein (PV) foci triggering AF.^5,6^ Superior vena cava isolation is feasible but limited by the drawbacks of thermal energies (radiofrequency or cryoablation) with a risk of lesion of adjacent structures (sinus node and phrenic nerve).^7–12^ As a consequence, the clinical benefit of SVC isolation in AF patients remains uncertain.^13–16^

Due to its tissue-selectivity, PFA could represent a valuable option to perform efficient and safe SVC isolation, reducing the risk of iatrogenic sinus node dysfunction (SND) or phrenic nerve palsy (PNP).^17^ Efficacy and safety of SVC isolation using PFA have been recently proven in an animal model,^18,19^ but evidence in humans is limited to rare case reports.

Hence, the goal of the present study was to evaluate the feasibility and safety of SVC isolation using a pentaspline PFA catheter, in patients undergoing AF catheter ablation.

## Methods

### Study population

From June 2023 to December 2023, consecutive patients undergoing PFA-based paroxysmal or persistent AF catheter ablation with SVC isolation in two high-volume academic centres were prospectively included. A written informed consent was obtained from each patient before the procedure, encompassing an off-label use of the Farapulse™ system (Boston Scientific, USA). Approval for this study was obtained from the local ethics committee and was in accordance with the Declaration of Helsinki.

### Atrial fibrillation catheter ablation

All procedures were performed using an institutional standardized workflow. All patients received direct oral anticoagulant for at least 4 weeks before the procedure and underwent computed tomographic scan and/or transoesophageal echocardiography within 48 h of the procedure to rule out atrial thrombus. All procedures were performed under general anaesthesia with uninterrupted oral anticoagulation. Administration of curare was at the discretion of the anaesthesiologist. Intravenous heparin was infused to maintain the activated clotting time above 300 s. A steerable quadri- or decapolar catheter was positioned in the coronary sinus (CS), if needed. Transseptal puncture was performed under fluoroscopic and/or transoesophageal echocardiograph guidance. An over-the-wire 31 or 35 mm–wide pentaspline PFA catheter (Farawave™, Farapulse, Boston Scientific, USA) was used in every patient, with a 0.035 inch J-tip guidewire (Cook Medical, USA). Pulmonary vein isolation was performed in all patients, followed by PWI in persistent AF patients at the operator’s discretion. Direct current cardioversion (DCC) was then performed if sinus rhythm was not restored. Additional PFA application was performed in case of non-isolated pulmonary vein or posterior wall, at the operator’s discretion.

### Superior vena cava isolation workflow

**Figure 1:**
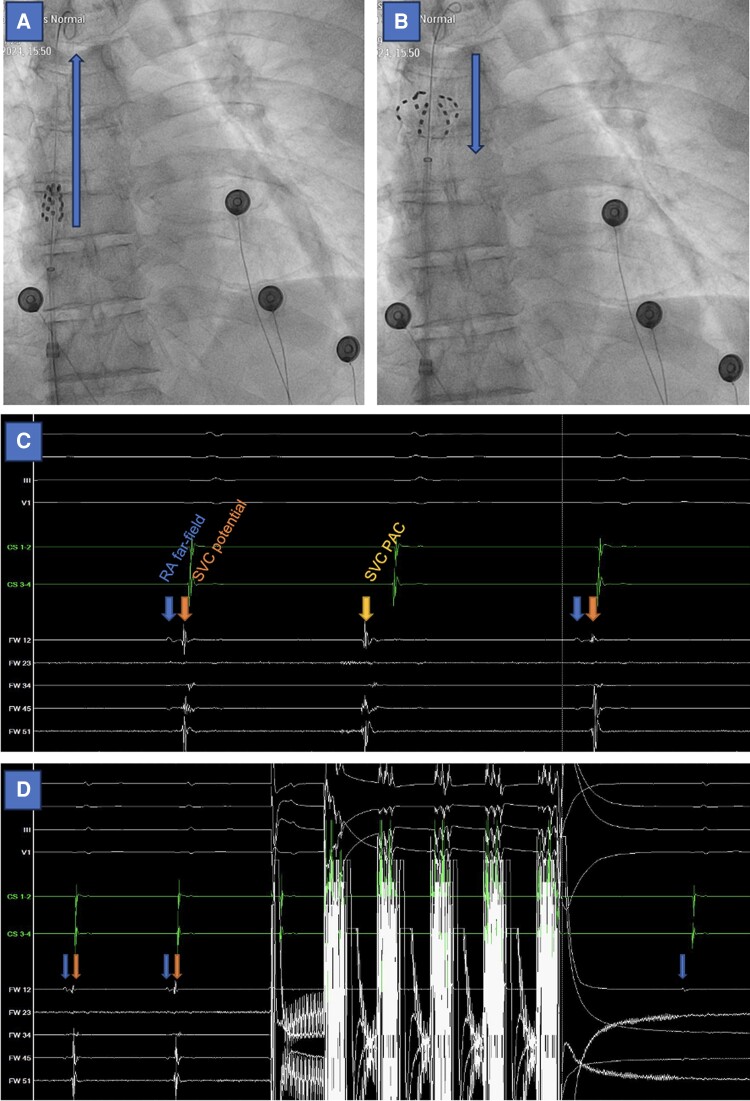
Superior vena cava isolation workflow. *(A*) Over-the-wire undeployed pentaspline PFA catheter (Farawave™, Boston Scientific, USA) in the SVC. (*B*) Deployment of the pentaspline PFA catheter in the SVC and slow withdrawal towards RA. (*C*) From *top* to *bottom*: surface ECG, CS catheter, and Farawave™ catheter. Distinct RA far-field (blue arrow) and SVC potential (orange arrow) can be seen, as well as a PAC originating from the SVC (yellow arrow). (*D*) From *top* to *bottom*: same as *(C)*. First PFA application and subsequent SVC isolation. PAC, premature atrial contraction; PFA, pulsed-field ablation; RA, right atrial; SVC, superior vena cava.

After PVI (±PWI and DCC), the sheath and the pentaspline catheter were withdrawn to the right atrium. During SVC isolation, the sheath was left at the inferior vena cava–right atrial junction. As illustrated in *Figure 1*, the J-tip guidewire was positioned in the high SVC. The pentaspline catheter was pushed over the wire into the high SVC and then deployed in a basket position until slight resistance. The pentaspline catheter in basket position was then slowly withdrawn until two distinct electrograms could be seen: right atrium far-field electrogram and SVC near-field electrogram. Phrenic nerve pacing using the pentaspline PFA catheter was performed before and after PFA applications and monitored by palpation. In addition, patients were monitored using right diaphragmatic compound motor action potential (CMAP), as previously described.^8^ Heart rate was monitored before and after PFA applications. Pulsed-field ablation applications were performed in a basket position, with rotation between each pair of applications. The total number of PFA applications was at the discretion of the operator, with a minimum of four applications and a maximum of eight applications. No atropine was used before or after PFA applications. For illustrative purposes, electroanatomical mapping was performed in four patients using the Orion™ multipolar catheter with the Rhythmia™ HDx system (Boston Scientific, USA).

### Post-procedural management

All patients underwent continuous telemetry monitoring for at least 24 h after the procedure and were discharged after overnight observation in the absence of complication. Class I and III antiarrhythmic drugs (AADs) were discontinued at discharge at the operator’s discretion. Outpatient follow-up was performed at 3 months, with clinical evaluation and 12-lead electrocardiogram. In case of symptoms suggestive of SND and/or PNP, 24-h Holter and/or deep-inspiration chest X-ray was performed.

### Outcomes

The acute feasibility outcome was the success of SVC isolation during the procedure, defined as the persistence (>15 min) of both entrance and exit blocks confirmed using the pentaspline catheter.

The acute safety outcomes were the incidence of PNP or SND after SVC isolation. Acute phrenic nerve stunning (PNS) was defined as the drop of CMAP amplitude > 30% from baseline. Acute PNP was defined as a loss of diaphragmatic contraction at the end of the procedure. In case acute PNP was diagnosed, deep-inspiration chest X-ray was performed at Day +1. Additionally, the first 20 consecutive patients of the study underwent systematic deep-inspiration chest X-ray at Day +1. Acute SND was defined as complete sinus arrest during procedure and until discharge.

### Statistical analysis

Data are presented as mean ± standard deviation or median [quartile 1 (Q1); quartile 3 (Q3)], as appropriate. Categorical variables are given as number of subjects with the attribute (percentage). For continuous variables, a Student’s *t*-test or a Mann–Whitney *U* test was performed, as appropriate. A *P* < 0.05 denoted statistical significance. Analyses were conducted using IBM SPSS Statistics for Macintosh (Version 23.0, IBM, Chicago, IL, USA).

## Results

### Baseline and procedural characteristics

Between June and December 2023, 105 patients (62.4 ± 8.4 years old, 85.7% male) underwent PFA-based AF catheter ablation with SVC isolation. Sixty-seven (63.8%) patients had persistent AF, and the mean CHA_2_DS_2_-VASc score was 2 ± 1 points. Baseline characteristics of the patients are detailed in *Table 1*. Procedural characteristics are detailed in *Table 2*. Median sinus cycle length was 901 (715; 1102 ms) at the start of the procedure.

**Table 1:** Baseline characteristics of the study population (*n* = 105)

Age, years	62.4 ± 8.4
Male gender, *n* (%)	90 (86)
Persistent AF, *n* (%)	67 (64)
Previous AF catheter ablation, *n* (%)	5 (4.8%)
BMI, kg/m^2^	26.8 ± 4.2
Hypertension, *n* (%)	50 (48)
Diabetes, *n* (%)	15 (14)
Heart failure, *n* (%)	35 (33)
LVEF, %	49 ± 12
Stroke/TIA, *n* (%)	3 (2.8)
CHA_2_DS_2_-VASc score	2 ± 1
Class I AAD, *n* (%)	19 (18)
Class II AAD, *n* (%)	85 (81)
Class III AAD, *n* (%)	31 (30)
Class IV AAD, *n* (%)	1 (1.0)
ACE-I/ARB, *n* (%)	20 (19)
ARNI, *n* (%)	12 (11)
iSGLT2, *n* (%)	25 (24)
MRA, *n* (%)	11 (10)
DOA, *n* (%)	100 (95)
VKA, *n* (%)	5 (4.8)
Pacemaker/defibrillator, *n* (%)	10 (10)

AAD, antiarrhythmic drug; ACE-I, angiotensin-converting enzyme inhibitor; AF, atrial fibrillation; ARB, angiotensin receptor blocker; ARNI, angiotensin receptor neprilysin inhibitor; BMI, body mass index; DOA, direct oral anticoagulant; iSGLT2, SGLT2 inhibitor; LVEF, left ventricular ejection fraction; MRA, mineralocorticoid receptor antagonist; TIA, transient ischemic attack; VKA, vitamin K antagonist.

**Table 2:** Procedural characteristics (*n* = 105)

Catheter size 31 mm, *n* (%)		94 (89)
Initial rhythm, *n* (%)	AF	57 (54)
	Sinus rhythm	44 (42)
	AT/AFl	4 (3.8)
Successful PVI, *n* (%)		105 (100)
Successful PWI, *n* (%)		46 (100)^a^
DCC, *n* (%)		45 (43)
Procedure duration, min		50 ± 15
LA dwell time, min		36 ± 13
Fluoroscopy time, min		11 ± 6

AF, atrial fibrillation; AFl, atrial flutter; AT, atrial tachycardia; DCC, direct current cardioversion; LA, left atrial; PVI, pulmonary vein isolation; PWI, posterior wall isolation.

^a^Forty-six patients underwent PWI.

### Feasibility

**Figure 2:**
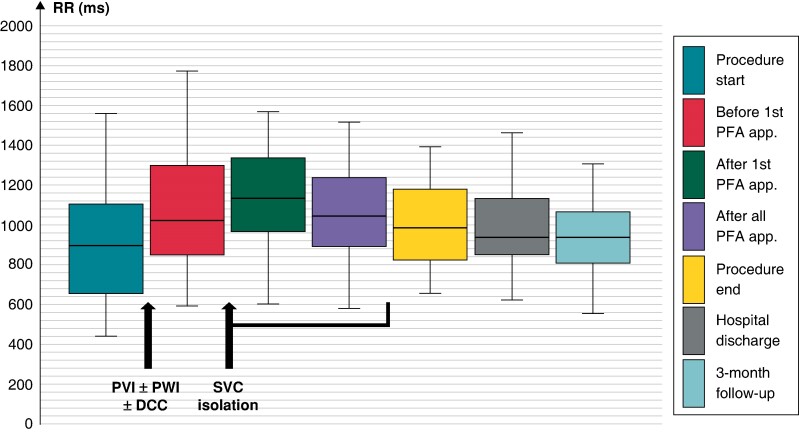
Illustrative example of pre- and post-SVC isolation using the pentaspline PFA catheter, with an electroanatomical mapping system. Right atrial voltage mapping in sinus rhythm (0.05–0.5 mV). Left panel, pre-SVC isolation; right panel, post-SVC isolation. White dashed lines represent SVC anatomy. DCC, direct current cardioversion; PFA, pulsed field ablation; PVI, pulmonary vein isolation; PWI, posterior wall isolation; SVC, superior vena cava.

Feasibility outcomes are detailed in *Table 3*. During catheter deployment, mechanical premature atrial contractions (PACs) originating from the SVC were seen in 86/105 (82%) patients. Complete SVC isolation was achieved in all patients. *Figure 2* is an illustrative example of pre- and post-SVC isolation using electroanatomical mapping. Mean duration of the SVC isolation workflow was 3.0 ± 1.3 min.

**Table 3:** Feasibility and safety outcomes

Feasibility outcomes	
SVC isolation after first PFA application	105 (100)
Total number of applications	6 ± 1
SVC entrance block, *n* (%)	105 (100)
SVC exit block, *n* (%)	105 (100)
Safety outcomes	
Phrenic nerve stunning, *n* (%)	67 (64%)
Phrenic nerve palsy, *n* (%)	0 (−)
Transient high degree SND, *n* (%)	5 (4.7)
Transient high degree AVB, *n* (%)	2 (1.9)

AVB, atrioventricular block; SND, sinus node dysfunction; SVC, superior vena cava.

### Acute safety outcomes

**Figure 3:**
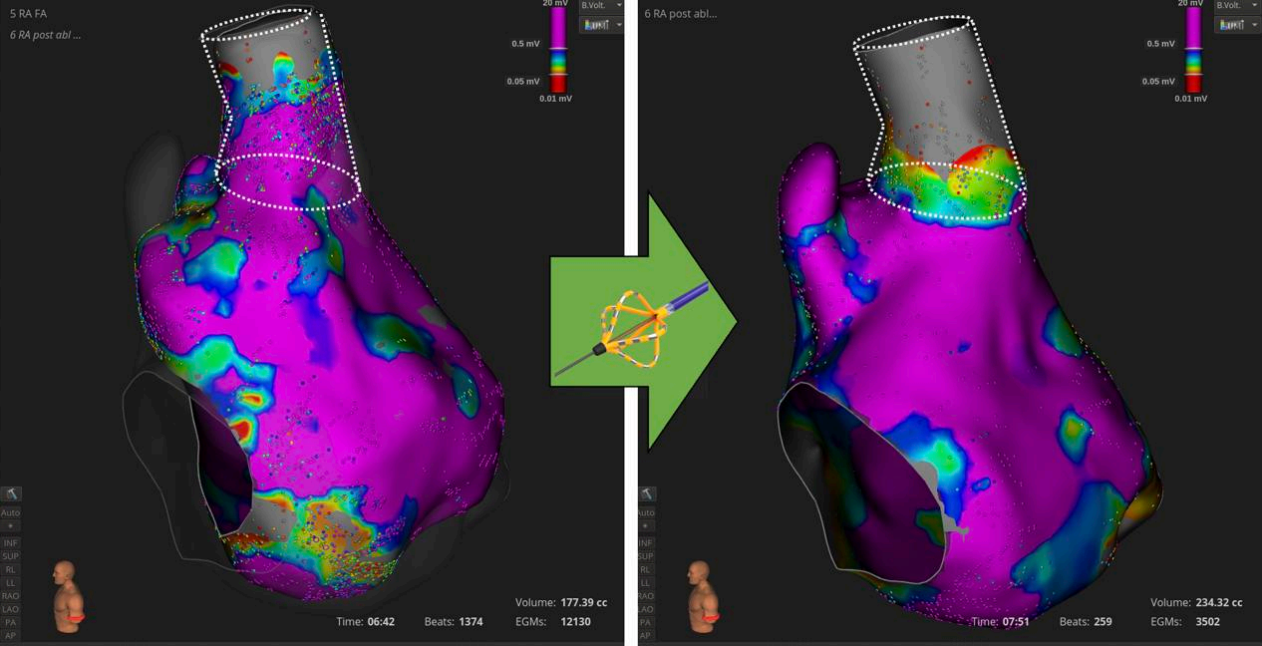
Safety outcome regarding sinus node function. Sinus node function (RR interval in ms; median; quartiles 1 and 3; and minimum and maximum values) before and after SVC isolation workflow. DCC, direct current cardioversion; PFA, pulsed-field ablation; PVI, pulmonary vein isolation; PWI, posterior wall isolation; SVC, superior vena cava.

Acute safety outcomes are detailed in *Table 3* and *Figure 3*. During the procedure, 67/105 patients (64%) experienced transient PNS. In those patients, the mean duration of PNS was 4.2 ± 2.5 min. At the end of the procedure, no patients experienced PNP, and systematic chest X-ray at Day +1 found no evidence of PNP.

Median sinus cycle length was 1020 (871; 1250) ms before the first PFA application, and 1133 (970; 1333), 1045 (893; 1218), and 984 (827; 1177) ms, respectively, after the first PFA application, after all PFA applications, and at the end of the procedure (*P* > 0.05). During SVC isolation workflow, 7/105 (6.7%) patients experienced transient conduction disturbances immediately after the first PFA application, as detailed in *Table 3*. At hospital discharge, median sinus cycle length was 938 (857; 1132) ms, with no recurrence of high-degree conduction disturbance.

No PFA-induced implantable cardioverter-defibrillator or pacemaker dysfunction occurred in the 10 patients with a cardiac implantable electronic device.

### Safety outcomes at 3-month follow-up

At the 3-month follow-up visit, no patient was lost to follow-up and 7/105 (6.7%) patients were using Class I or III AAD. Median sinus cycle length was 952 (816; 1044) ms (*P* > 0.05 vs. before the first PFA application). There was no occurrence of symptomatic SND or PNP. No chronic ICD/PM dysfunctions were observed.

## Discussion

The main results of this study can be summarized as follows: SVC isolation using a pentaspline PFA catheter is feasible and safe in patient undergoing paroxysmal or persistent AF catheter ablation.

Pulmonary vein isolation is the cornerstone of AF catheter ablation, but approximately a third of patients might present non-PV triggers, especially in the SVC.^5^ Catheter ablation of ectopic beats triggering AF and originating from the SVC has been hypothesized to improve AF catheter ablation efficacy.^6^ As a consequence, SVC isolation has been routinely performed during the previous two decades using thermal energies (i.e. radiofrequency or cryoablation) in selected patients.^7–11^ However, thermal energies remain associated with a risk of collateral damage to adjacent structures such as the sinus node and the right phrenic nerve, with a subsequent risk of SND and/or PNP.^7–11,13^ An additional risk of SVC stenosis has been hypothesized but never confirmed. Recently, PFA has emerged as a non-thermal energy with an optimal efficiency–safety profile to perform PVI.^1,2^ The cardiomyocyte-specificity of PFA could limit the risk of collateral damage to adjacent structures.^17^ A recent animal study found that PFA can effectively isolate SVC (with transmural tissue damage) without PNP or SND,^18,19^ and, until known, only few case reports supported the use of PFA to isolate SVC in humans.

In the present study, including consecutive patients undergoing AF catheter ablation, we found that SVC isolation using a pentaspline PFA catheter is feasible and efficient. All SVCs were isolated after the first PFA application, with shorter workflow duration than previously reported with thermal energies.^11^ Our findings are in line with recent animal studies, in which PFA-based SVC isolation induced a transmural lesion with complete and durable SVC isolation (confirmed by remapping at 3 to 4 weeks).^18,19^

Previous studies in humans found a high rate of complications induced by thermal energy–based SVC isolation. Chen *et al.*^12^ found that 4.5% of patients experienced SND after radiofrequency-based SVC isolation. This could be explained by the close proximity between sinus node and SVC ostium and by the potential collateral damage to the sinus node artery. Cryoablation might offer a better safety profile, with only transient SND in 7.7% of patients undergoing cryoablation-based SVC isolation.^9,10^ In the present study, we found no evidence of SND after the procedure and up to 3-month follow-up. Seven patients (6.7%) experienced transient conduction disturbances (in the sinus node but also the atrioventricular node). This phenomenon could be due to ganglionated plexi stimuli, as it is frequently observed during PFA-based PVI,^20^ even though direct nodal stunning cannot be excluded.

Phrenic nerve palsy has been reported in 2.1–9.2% of patients undergoing SVC isolation, respectively with cryoablation and radiofrequency.^7,9^ Even though PFA is considered as a myocardial-specific energy,^17^ alterations of phrenic nerve function have been reported previously. In the MANIFEST-17K registry, the incidence of PNS was 0.06%, but this is likely to be underestimated by a registry-based study design without systematic assessment of phrenic nerve function (unpublished data by Reddy V *et al.*, American Heart Association Congress 2023). Recently, Schmidt *et al.*^1^ reported that phrenic nerve dysfunction was present in 0.32% of patients undergoing PFA-based PVI (three PNS and one PNP). In our study, we found no evidence of persistent PNP, although transient PNS was frequent immediately after PFA applications. This is in line with a recent animal study from Howard *et al.*,^19^ who found that PNS was frequent and dose-dependent when PFA was delivered in proximity to the phrenic nerve in the SVC. Notwithstanding, the authors found no evidence of chronic PNP, which is also in line with our results. The physiopathology of PNS is likely to be due to an electrophysiological mechanism rather than a thermal injury, as they found no anomaly on phrenic nerve histopathology. Altogether, those data support a reasonable safety profile of PFA-based SVC isolation, with the need for an ongoing vigilance regarding the effect of PFA on phrenic nerve function.

The impact of PFA-based SVC isolation on AF catheter ablation efficacy is currently unknown. Previous randomized controlled trial (RCTs) using thermal energies failed to prove a consistent superiority of adding systematic SVC isolation on top of PVI during a first procedure.^13–16^ Our study adds some new insights to explain those findings: we found evidence of SVC activity in 82% of patients admitted for AF catheter ablation and undergoing SVC isolation. A corroborative would be that approximately 20% of patients undergoing AF catheter ablation might not benefit from empirical SVC isolation, which might have underestimated the real effect of SVC isolation in previous RCTs. This hypothesis is corroborated by recent findings from Dong *et al.*^14^ who found that electroanatomical mapping–guided SVC isolation, in addition to PVI, did not increase the success rate of paroxysmal AF catheter ablation in patients who had no identifiable SVC triggers. Altogether, those data support the need for a dedicated RCT to study the benefit of adding targeted PFA-based SVC isolation on top of PVI, in patients with evidence of SVC activity during AF catheter ablation.

### Limitations

Our study might suffer from several limitations. Regarding phrenic nerve function, systematic chest X-ray was only performed in the first 20 consecutive patients, which might have underestimated the incidence of PNP. However, no loss of diaphragmatic contraction was observed at the end of the procedure, and there was no evidence of symptomatic PNP at the 3-month follow-up visit. Regarding SND, systematic 24-h Holter monitoring to assess sinus node function was not performed in our study, which might have underestimated the incidence of SND. However, post-procedural telemetry monitoring was performed until discharge and found no evidence of durable SND. Moreover, 3-month systematic Holter monitoring would be likely to find incidental SND, as it is highly prevalent in patients with AF.^21^ In this case, SND would not be related to SVC isolation, but rather with atrial cardiomyopathy. Regarding a potential risk of SVC stenosis, we did not perform systematic assessment of SVC size. However, due to its non-thermal nature, there is currently no report of PV stenosis after PFA-based PVI.^22^ None of the patients had evidence of SVC syndrome at the 3-month follow-up visit; further follow-up is required to confirm this point. Finally, regarding the durability of PFA-based SVC isolation, we did not perform systematic remapping of the SVC at 3 months to confirm isolation. During follow-up, one patient underwent a redo procedure for recurrent persistent AF, and remapping found complete and durable SVC isolation. Current evidence supporting the durability of PFA-based PVI might be extrapolated to PFA-based SVC isolation.^23^

## Conclusions

Superior vena cava isolation using a pentaspline PFA catheter is feasible and safe. The impact of PFA-based SVC isolation on AF catheter ablation efficacy and the assessment of the durability of PFA-based SVC isolation require dedicated trials.

## Data Availability

All relevant data are within the manuscript.
